# Valproic Acid Inhibits Proliferation and Reduces Invasiveness in Glioma Stem Cells Through Wnt/β Catenin Signalling Activation

**DOI:** 10.3390/genes9110522

**Published:** 2018-10-26

**Authors:** Gabriele Riva, Chiara Cilibrasi, Riccardo Bazzoni, Massimiliano Cadamuro, Caterina Negroni, Valentina Butta, Mario Strazzabosco, Leda Dalprà, Marialuisa Lavitrano, Angela Bentivegna

**Affiliations:** 1 School of Medicine and Surgery, University of Milano-Bicocca, via Cadore 48, 20900 Monza, Italy; gabrieleriva1986@gmail.com (G.R.); c.cilibrasi@campus.unimib.it (C.C.); r.bazzoni@campus.unimib.it (R.B.); massimiliano.cadamuro@unimib.it (M.C.); caterina.negroni@plymouth.ac.uk (C.N.); v.butta@gmail.com (V.B.); mario.strazzabosco@unimib.it (M.S.); leda.dalpra@unimib.it (L.D.); marialuisa.lavitrano@unimib.it (M.L.); 2 NeuroMI, Milan Center of Neuroscience, Department of Neurology and Neuroscience, University of Milano-Bicocca, San Gerardo Hospital, via Pergolesi, 20900 Monza, Italy; 3 Ph.D. Program in Neuroscience, University of Milano-Bicocca, via Cadore 48, 20900 Monza, Italy; 4 Department of Neurology and Neurosurgery, Montreal Neurological Institute and Hospital, McGill University, Montreal, QC H3A 2B4, Canada; 5 International Center for Digestive Health, University of Milano-Bicocca, 20126 Milano, Italy; 6 Schools of Medicine and Dentistry, University of Plymouth, Plymouth, Devon PL6 8BT, UK; 7 Liver Center, Section of Digestive Diseases, Yale University School of Medicine, New Haven, CT 06510, USA

**Keywords:** glioma stem cells, valproic acid, Wnt/β-catenin signalling pathway, cell proliferation, cell invasion

## Abstract

Glioblastoma is the most common malignant brain tumour in adults. The failure of current therapies can be ascribed to glioma stem cells (GSCs), which can rapidly repopulate the tumour following the initial treatment. The study of histone deacetylase inhibitors, such as valproic acid (VPA), is becoming an attractive field in cancer research. However, the exact mechanisms underlying its anti-cancer effect remain to be elucidated due to its pleiotropic effects on several cell-signalling pathways. Ingenuity Pathway Analysis (IPA) bioinformatics analysis was performed on genome-wide data regarding GSCs methylome to identify the signalling pathways mainly affected by methylation changes induced by VPA. Real time PCR and luciferase reporter assay were used to better investigate VPA effects on Wnt/β-catenin signalling pathway. VPA effect on GSC proliferation was evaluated by 3-(4,5-Dimethylthiazol-2-yl)-2,5-Diphenyltetrazolium Bromide (MTT) and Trypan blue assays. Finally, VPA impact on GSC motility was demonstrated by Boyden chamber assay and further confirmed evaluating the expression levels or localisation, through western blot or immunofluorescence, of Twist1, Snail1, E-Cadherin and N-Cadherin. The bioinformatics analyses performed on GSCs methylome highlighted that Wnt/β-catenin signalling was affected by the methylation changes induced by VPA, which could influence its activation status. In particular, we pointed out a general activation of this pathway after VPA exposure, which was accompanied by an inhibitory potential on GSCs proliferation. Finally, we also proved VPA’s ability to inhibit GSCs invasion through Snail1 and Twist1 downregulation and E-Cadherin relocalisation. VPA treatment may represent a new, interesting therapeutic approach to affect GSC proliferation and motility, but further investigations are certainly needed.

## 1. Introduction

Glioblastoma (GBM) is the most common and aggressive type of primary brain tumour. Despite several biological and clinical studies, the median survival of GBM patients is still about 15 months post-diagnosis, with high risk of recurrence [1]. Increasing evidence has suggested that GBM contains a small subpopulation of tumour cells with stem-like properties, called “glioma stem cells” (GSCs) [2,3,4,5], characterised by self-renewal capacity, multilineages differentiation potential, enhanced invasive behaviour and tumorigenicity in vivo. Intriguingly, GSCs have been indicated as one of the major causes for tumour relapse after conventional treatments because of their strong chemo- and radio-resistance [6]. Thus, new therapeutic strategies are needed to completely eradicate this tumour.

Epigenetic mechanisms have being exploited as new potential therapeutic targets and increasing evidence suggests that epigenetic aberrations, apart from genetic alterations, may play a key role in the development and/or progression of gliomas [7]. Therefore, the reversibility of epigenetic alterations encourages the use of epigenetic drugs, such as histone deacetylase inhibitors (HDACi), as an attractive approach to “reset” the abnormal cancer epigenome [8].

Valproic acid (VPA), a short-branched fatty acid derived from naturally-occurring valeric acid, is an HDACi that can be used to control seizures in patients with brain tumours [9]. The VPA capability of prolonging overall survival in GBM patients, reported by some retrospective clinical studies [10], and its known pharmacological profile and moderate toxicity, encouraged the study of this drug in the context of a future therapy for the treatment of GBM. Accordingly, several in vitro and in vivo glioma studies shed light on its numerous potent anti-tumour effects [11,12,13]. However, the exact mechanisms underlying its anti-cancer effect remain to be elucidated, also in GSCs, due to its pleiotropic effects on diverse biological processes and cell signalling pathways.

Interestingly, it has been demonstrated that VPA is able to affect the canonical Wnt/β-catenin signalling pathway, a highly evolutionary conserved pathway with a key role in embryogenesis regulation and self renewal in stem cells [14,15], found aberrantly activated in a wide range of cancers [16,17]. Thus, several studies pointed out that the constitutive activation of Wnt/β-catenin signalling is able to promote tumourigenesis in different tissues [18,19,20,21]. This pathway is frequently impaired also in GBM, through either genetic or epigenetic alteration, [22] and its aberrant activation has been associated to gliomagenesis, GSCs self-renewal, and invasive capability [23,24,25]. However, the role of Wnt/β-catenin signalling is still not so clear in cancer cells, with both its augmentation and repression associated with pro- and anti-tumour effects [26,27,28,29]. In fact, recent studies yielded conflicting data on whether the hyper-activation or repression of Wnt signalling pathway may be a promising therapeutic strategy [30], and intriguingly, its activation has been associated with HDACi anti-tumour effects [31,32,33]. Anyhow, to date, there is no study of VPA effects on Wnt/β-catenin signalling pathway in GSCs.

Therefore, in this work, starting from some preliminary data concerning the effect of VPA on GSC methylome [7], we pointed out the ability of this drug to activate Wnt/β-catenin pathway. Interestingly, this activation was associated to a growth inhibition of all GSCs and to a selective repression of GSC-invasive behaviour, as demonstrated by Snail1 and Twist1 impairment, two crucial factors involved in the epithelial-mesenchymal transition (EMT) programme, and E-Cadherin relocation.

## 2. Materials and Methods

### 2.1. Cell Lines and Cell Culture Conditions

All the GSC lines (GBM2, GBM7, GBM04, G144, G179, G166, and GliNS2) were isolated from patients affected by GBMs previously characterised for their stemness properties [34,35]. GSCs and human foetal neural stem cell (CB660) expansion was carried out as described in [36].

### 2.2. Drug and Treatments

Sodium Valproate (Molecular Weight = 166.9 g/mol, Sigma-Aldrich, Saint Louis, MO, USA) was dissolved in distilled water to make a 500 mM stock solution, and then diluted to 2 mM with distilled water. The stock preparation was stored at −20 °C.

### 2.3. Bioinformatics Analysis by Ingenuity Pathway Analysis Software

The pathway analysis was generated using the Ingenuity Pathway Analysis software version 1.1 (IPA, Ingenuity System, Redwood City, CA, USA; www.qiagenbioinformatics.com/products/ingenuity-pathway-analysis). IPA software examines functional relationships within an input list of genes, and identifies the pathways from the IPA library of canonical pathways that were most significantly associated with the dataset. The significance of the association between the dataset and canonical pathways was measured in two ways: (1) a ratio of the number of molecules from the dataset that map on the pathway divided by the total number of molecules that map on the canonical pathway is displayed; (2) Fisher’s exact test was used to calculate a *p*-value determining the probability that the association between the genes in the dataset and the canonical pathway is explained by chance alone. Our datasets were derived from the experiments described in [7], in which we performed a genome-wide DNA methylation analysis on untreated and 2 mM VPA-treated cultures of GBM2 and G144 cells for 96 h. The input lists submitted to IPA software contained only those genes that changed their methylation status after treatment [7]. The predicted activation state of significantly-altered pathways was determined by a z-score algorithm that compared the dataset of genes that changed their methylation status after treatment with the expected canonical pathway patterns. 

### 2.4. RNA Extraction and cDNA Synthesis

RNA extraction from CB660, untreated and 2 mM VPA 96 h treated GSCs was performed using the miRNeasy Mini Kit (Qiagen, Hilden, Germany), according to the manufacturer’s protocol. RNA samples from CB660 and untreated GSCs were converted into first-strand cDNA using the RT^2^ First Strand Kit (Qiagen).

### 2.5. Real Time-PCR Array

RT^2^ Profiler PCR Arrays (Qiagen) were assessed on untreated and 2 mM VPA 96 h treated cells according to the manufacturer’s protocol, using a 96-well Wnt signalling pathway-specific PCR array, containing primers for 84 Wnt pathway-related genes, to perform a preliminary screening on two cell lines (GBM2 and G144). Then, we extended the analysis to all GSC lines using a 96-well PCR array custom containing primers for seven Wnt pathway selected genes (*WNT1*, *FZD4*, *CTNNB1*, *EP300*, *CREBBP*, *TCF7*, and *MYC*), and for 2 housekeeping genes (*HPRT1*, *TBP*).

The cut-off values for gene expression fold changes were established at ±1.5: values ≥ +1.5 indicate gene upregulation, while values ≤ −1.5 indicate gene downregulation. The gene expression fold changes data were obtained as mean values derived from at least two independent experiments.

### 2.6. Real Time PCR

GSCs baseline Wnt target gene expression levels and *AXIN2*, *CD44* and *DKK1* expression levels after 96 h VPA 2 mM treatment were assessed using the 5× hot firepol evagreen (Solis BioDyne, Tartu, Estonia), according to the manufacturer’s protocol. Glyceraldehyde 3-phosphate dehydrogenase (*GAPDH*) was used as a housekeeping gene. CB660 cells or untreated GSCs were used as normal controls. The primers used are reported in Table S1.

Quantitative PCRs were carried out using the ABI StepOne (Applied Biosystems, Foster City, CA, USA), according to the manufacturer’s instructions. Melt curve analysis was performed to confirm specificity of amplified products. The cut-off values for gene expression fold changes were established at ±1.5: values ≥ +1.5 indicate gene upregulation, while values ≤ −1.5 indicate gene downregulation. The gene expression data were obtained as mean values derived from two independent experiments.

### 2.7. Luciferase Reporter Assay

GBM2 cells were plated in 96-well plates and transiently co-transfected by means of lipofectamine 3000 (Thermo Fisher Scientific, Waltham, MA, USA) with T cell factor (TCF) reporter plasmid (TOP) or mutated TCF reporter plasmid (FOP) and Renilla thymidine kinase-luciferase vector, used to normalise the transfection efficacy. Forty-eight hours after the transfection, cells were treated with VPA 2 mM for 96 h. Afterwards, the activities of firefly luciferase and Renilla luciferase were measured in controls and treated samples using the fluorescence microplate reader (infinite M2000pro, Tecan, Mannedorf, Switzerland) with a Dual-Glo luciferase assay system (Promega, Fitchburg, WI, USA), according to the manufacturer’s instructions. The TOP and FOP-Flash reporter activity is presented as the relative ratio of firefly luciferase activity to Renilla luciferase activity in treated cells compared to matching controls. Experiments were performed at least three times in triplicate.

### 2.8. MTT Assay

Cell metabolic activity was assessed by the MTT (3-[4,5dimethylthiazol-2-yl]-2,5-diphenyl tetrazolium bromide), Sigma-Aldrich by Merck, Darmstadt, Germany) assay, as already described in [37], after exposure to VPA at various concentrations (0.5, 1 and 3 mM). The percentage of inhibition was determined by comparing the absorbance values of drug-treated cells with that of untreated controls: [(treated-cell absorbance/untreated cell absorbance) × 100]. The results reported are the mean values of at least three different experiments performed in quadruplicate.

### 2.9. Trypan Blue Dye Exclusion Assay

Cells were plated in 60 mm Petri dishes at a density of 1.2 × 10^6^ cells/dish and cultured overnight. Then, cells were treated with 2 mM VPA for 96 h and stained using the trypan blue dye (Sigma-Aldrich by Merck, Darmstadt, Germany) to count live and dead cell numbers and determine the effect of VPA on the proliferation rate. The treated samples were compared with the untreated controls. The results reported are the mean values of four different experiments.

### 2.10. Boyden Chamber Assay

This assay was performed using a Boyden chamber with a gelatin-coated polycarbonate filters with 8 μm pore size (NeuroProbe, Gaithersburg, MD, USA). Briefly 5 × 10^3^ cells, untreated or treated with 2 mM VPA for 96 h, were seeded in the upper compartment of the chamber with serum-free medium. Medium with 10% FBS (EuroClone, Milano, Italy) was added into the lower compartment. After 24 h of culture at 37 °C, cells that did not migrate were removed from the upper face of the filters, while cells on the lower surface of the membrane were fixed in methanol and stained with eosin G and tetrazolium blue chloride. Photographs were taken and the number of migrated cells was quantified using Image J software. The experiments were performed in triplicate.

### 2.11. Protein Extracts and Western Blotting

Cells were treated for 96 h with 2 mM VPA, and at the end of the treatment, equal concentrations of cell lysate from nuclear fractions were obtained as already described [38], were electrophoresed on a 4–12% NuPAGE Novex Bis-Tris gel (Life Technologies by Thermo Fisher Scientific, Waltham, MA, USA) with MES (NuPage Novex, Life Technologies by Thermo Fisher Scientific, Waltham, MA, USA). Western blots were performed using standard procedures [38]. Primary antibodies used were: goat anti-Snail1 (1:1000, AbCam, Cambridge, UK), and rabbit anti-Twist1 (1:500, Santa Cruz Biotechnology, Dallas, TX, USA). As loading control protein, rabbit anti-Histone 3 (1:2000, Sigma-Aldrich) was used. Proper HRP-conjugated secondary antibodies were used (1:2000 anti-rabbit, Bio-Rad, Hercules, CA, USA, 1:5000 anti-goat Santa Cruz Biotechnology). Proteins were visualised using SuperSignal West Pico or Dura chemiluminescent substrate (Thermo Fisher Scientific, Waltham, MA, USA) with a Kodak Image Station 440 CF (Eastman Kodak Co., New Haven, CT, USA). Bands were then quantified with ImageJ (https://imagej.nih.gov/ij/) and results were normalised versus controls. The experiments were performed at least in triplicates.

### 2.12. Immunofluorescence

Immunofluorescence (IF) assay was performed on untreated and 96 h VPA 2 mM treated GBM04, GBM2, GBM7 and G144 cells, using the following antibodies: mouse anti-N Cadherin (Zymed by Thermo Fisher Scientific, 1:200) and mouse anti–E Cadherin (BD Biosciences, San Jose, CA, USA 1:50) primary antibodies, anti-mouse secondary fluorescent antibody (Life technologies by Thermo Fisher Scientific, 1:2000). Phalloidin (Life technologies by Thermo Fisher Scientific, 1:40) has been used for cytoskeleton visualisation. Coverslips were mounted using the Vectashield mounting solution, containing DAPI (Vector laboratories, Burlingame, CA, USA), for nuclei visualisation. Representative images were acquired using a fluorescent microscope.

### 2.13. Statistical Analysis

Statistical analysis was carried out performing *t*-test on raw data, by means of Excel spreadsheet (Microsoft Office 2013, Microsoft Corporation, Redmont, WA, USA). The critical level of significance was set at *p* < 0.05.

## 3. Results

### 3.1. Valproic Acid Induced DNA Methylation Changes in Wnt Pathway-Related Genes

In a previous work, we performed a genome-wide DNA methylation analysis on two GSC lines (GBM2 and G144) after exposure to 2 mM VPA for 96 h, demonstrating its ability to induce deep changes, not only in histone acetylation, but also in the methylation pattern of these cells [6].

In the present work, data from genome-wide DNA methylation analysis were submitted to IPA software to identify target molecular pathways that may have been affected. First of all, it is clear that in both cell lines, the methylation shift induced by VPA involved multiple molecular pathways. Among others, one of the pathways affected by methylation changes in both the cell lines was the Wnt signalling pathway. Interestingly, with regards to the GBM2 cell line, Wnt signalling pathway modulation by VPA was shown explicitly by IPA analysis (Figure S1), while in the G144, this was proven through the presence of a more generic “Glioblastoma multiforme signalling” (Figure S2A), which also includes the Wnt signalling pathway (Figure S2B). Z-score values, calculated by IPA through an algorithm that compared the dataset of genes that changed their methylation status after treatment with the expected canonical pathway patterns, gave us a prediction of the activation state of the pathways affected by methylation changes after VPA exposure. Negative and positive z-scores are associated, respectively, to a predicted inactivation and activation of a specific pathway. In particular, with regard to the Wnt signalling pathway, GBM2 showed a negative z-score, while G144 showed a positive z-score, indicating, respectively, a predicted, but only hypothetical, inactivation or activation of this pathway after VPA treatment (Figures S1 and S2).

Therefore, we then focused our attention on the Wnt/β-catenin signalling pathway, deepening the effect of VPA on its activation status, as its aberrant activation has been associated with GBM development and progression. Moreover, our previously-published data on genome-wide analysis had shown that several Wnt pathway-related genes were strongly affected by copy number alterations (CNAs) in our GSC lines (Table S2), suggesting that Wnt pathway deregulation could play a key role in the regulation of GSC biology [21]. In particular, 14 out of 30 Wnt signalling pathway-related genes (about 50%) reported a CNA in at least one cell line, and a total of 25 CNAs involving these genes were registered in our GSC lines (Table S2). Therefore, on the basis of all these preliminary data, we thought that a deeper investigation of the VPA effect on this pathway might be crucial.

### 3.2. Valproic Acid Activated the Wnt Signalling Pathway in GSCs

In order to better evaluate the effects of VPA on this molecular pathway and its predicted activation or inactivation, we performed a preliminary screening on the expression of 84 Wnt-related genes using RNAs from untreated and 96 h VPA-treated GBM2 and G144 cells. As reported in Table 1, VPA was able to sharply modulate the transcription of several genes in both cell lines. In particular, GBM2 and G144 cell lines showed changes in the expression levels of 39 and 56 out of 84 genes, respectively. Among these, 27 genes showed the same alteration in both the cell lines after VPA exposure, while nine genes presented no alteration.

Therefore, starting from these preliminary data, we decided to extend our analysis to the other five GSC lines, investigating just a selection of ten Wnt-related genes (*WNT1*, *FZD4*, *CTNNB1*, *EP300*, *CREBBP*, *TCF7*, *MYC*, *AXIN2*, *CD44*, and *DKK1*). Some of these genes encode for proteins which carry out their function at different levels of the signalling cascade; others, such as AXIN2, CD44 and DKK1, can be considered specific targets of the Wnt signalling pathway, as demonstrated in [24], and can be used to assess the activation status this pathway. Among these, seven genes were already investigated in the previously-analysed 84 Wnt-related genes panel. Six out of seven genes showed the same alterations or no alterations in the expressions levels after VPA, while *MYC* presented different behaviour in the two cell lines taken into consideration.

**Figure 1:**
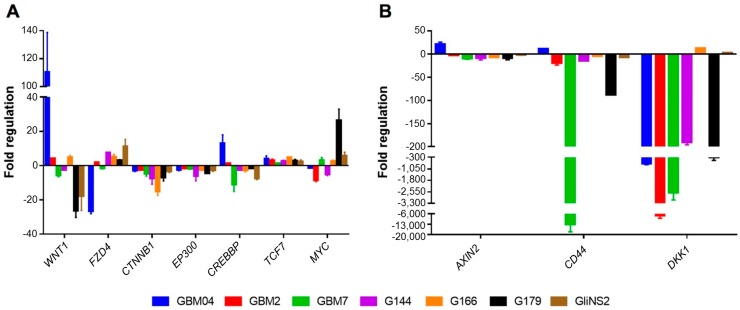
Glioma stem cells (GSCs) show a general basal inactivation of the Wnt/β-catenin signalling pathway. Basal expression levels of seven Wnt signalling related (**A**) and target genes (**B**) were evaluated in seven GSC lines. *GAPDH* gene was used as control of constitutive expression. Calculations of relative expression were performed with ∆∆C_t_ method, using CB660 cells as reference. Bars represent standard error of the mean (SEM).

First of all, the Wnt-related genes basal expression was evaluated in all the seven GSC lines (Figure 1A). When compared to foetal neural stem cells (CB660), GSCs showed variable expression levels of *WNT1* (ligand), *FZD4* (receptor) and the downstream oncogene *MYC*. *TCF7* (transcription factor) was upregulated in all the cell lines, while *CTNNB1*, the most important effector of the Wnt signalling pathway, *CREBBP* and *EP300*, two transcriptional coactivators functioning as histone acetyltransferases, were generally downregulated (except for GBM04, which showed an upregulation of *CREBBP*). Interestingly, the Wnt target genes *CD44*, *DKK1*, and especially *AXIN2*, showed a downregulation in most of the cell lines (Figure 1B). In particular, AXIN2 and CD44 were downregulated in all the cell lines except for GBM04, while DKK1 was downregulated in five out of seven cell lines. This demonstrated that Wnt signalling pathway seemed to have a predominant inactivation in GSCs when compared to foetal neural stem cells.

**Figure 2:**
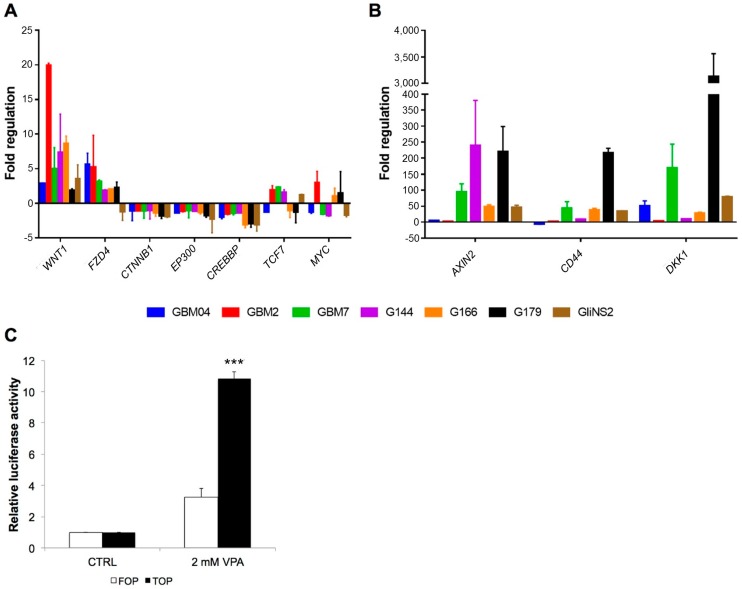
Valproic acid (VPA) induced an activation of the Wnt/β-catenin signalling pathway. (**A**) Expression variations of seven Wnt signalling related (**A**) and target genes (**B**) were evaluated in seven GSC lines, after 96 h of 2 mM VPA exposure. *HPRT*, *TBP* or *GAPDH* genes were used as control of constitutive expression. Calculations of relative expression were performed with ∆∆Ct method, using untreated GSCs as references. Bars represent SEM. (**C**) VPA exposure resulted in the increase of TopFlash luciferase activity, indicating the transcriptional activity of β-catenin/TCF complex. Bars indicate SEM. *** *p* < 0.001.

Afterwards, we evaluated the expression of the same ten genes after treatment with 2 mM VPA (Figure 2A,B). Results showed that VPA modulated Wnt signalling pathway genes in all the GSC lines (Figure 2A). In particular, *WNT1* and *FZD4* were upregulated in almost all the cell lines after 96 h of VPA treatment (except for *FZD4* in GliNS2), *CTNNB1* was downregulated in G179 and GliNS2, while in the other cell lines it showed no expression variations. *CREBBP* and *EP300* were downregulated in five out of seven and two out of seven cell lines, respectively. The transcription factor *TCF7* was upregulated in GBM2, GBM7 and G144 cells, while the downstream oncogene *MYC* showed a very heterogeneous expression among GSC lines after VPA administration; in particular, MYC was upregulated in GBM2 cell line and downregulated in GBM7, G144, and GliNS2, while in the other cell lines, the observed variations did not overcome the threshold value of −1.5. Finally, Wnt target genes showed an upregulation in all the cell lines (except for *CD44* in GBM04), highlighting that VPA is able to induce an activation of this pathway (Figure 2B).

This was further validated by a TOP/FOP flash reporter assay performed in GBM2 cell line, which showed that VPA significantly induced the transcriptional activity of β-catenin/TCF complex (Figure 2C).

### 3.3. Valproic Acid Reduced Glioma Stem Cells Proliferation

**Figure 3:**
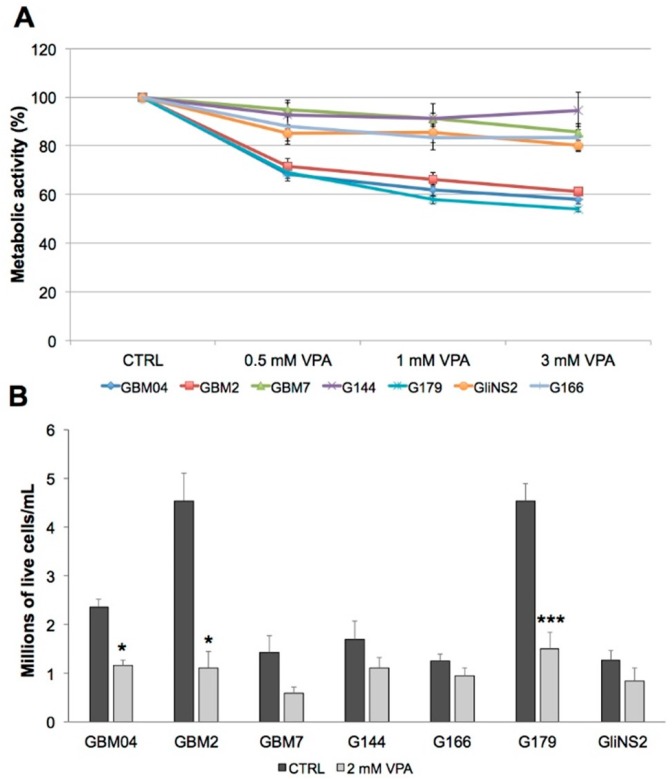
VPA inhibits GSCs proliferation. (**A**) Metabolic activity was analysed in seven GSC lines by MTT assay after 96 h exposure to escalating doses of VPA (0.5,1 and 3 mM). Bars represent SEM. For statistical analysis, see Table S1. (**B**) The proliferation rate was analysed by Trypan Blue dye exclusion assay after 96 h exposure to 2 mM VPA. Results are reported as the number of live cells/mL in treated samples compared to the matching untreated ones. Bars represent SEM. * *p* < 0.05; *** *p* < 0.001.

To assess if VPA could have an inhibitory effect on metabolic activity and proliferation, we performed an MTT assay in seven GSC lines after 96 h of treatment with different drug concentrations (Figure 3A, Table S3). VPA was able to induce a dose-dependent reduction of the metabolic activity in all the GSCs. However, this response was heterogeneous among our cell lines, with GBM2, G179, and GBM04 showing the maximum reduction of metabolic activity, i.e., 40%, compared to the other ones, which showed only a slight decrease. Indeed, GBM7, G166, and GliNS2 cell lines displayed only a 20% reduction of this parameter, and G144 cells were even less responsive, showing a 10% reduction.

These data were validated by Trypan blue assay using 2 mM concentration, the most useful VPA dose for future clinical application [20]. As shown in Figure 3B, after 96 h of treatment, VPA was able to slow down the proliferation rate in all the GSC lines. In particular, once again, GBM04, GBM2, and G179 showed the most relevant and statistically-significant reduction (45–80%) of the cell growth after treatment. A reduction of the proliferation rate was detected also in all the other cell lines, even if it was not statistically significant.

### 3.4. Valproic Acid Impaired Glioma Stem Cells Invasive Behaviour

**Figure 4:**
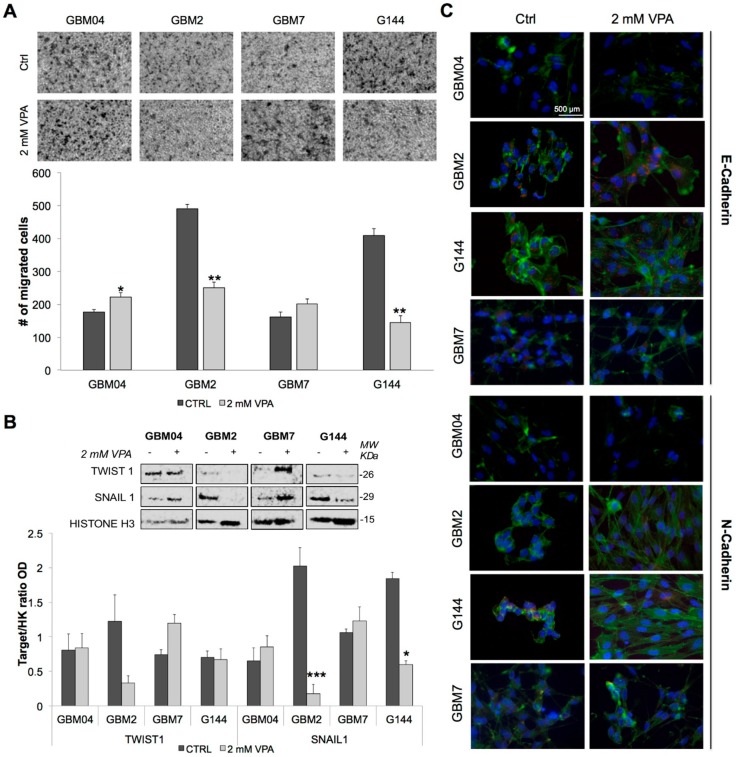
VPA selectively inhibit cell invasion. (**A**) Invasive behaviour was evaluated by Boyden chamber assay in four GSC lines after 96 h exposure to VPA 2 mM. Representative images are shown. Results are reported as the number of migrated cells in treated samples compared to the matching untreated ones. Bars represent SEM. * *p* < 0.05; ** *p* < 0.01. (**B**) Representative images and quantitative results of Western blot analysis on Twist1 and Snail1 in four GSC lines are reported. Protein expression levels were normalised on Histone H3. Values are expressed in Arbitrary Unit (AU). * *p* < 0.05; ** *p* < 0.01; *** *p* < 0.001. (**C**) Representative images of untreated or 96 h 2 mM VPA treated GSCs stained for E or N-Cadherin (red), phalloidin (green) and DAPI (blue) are reported.

Since the involvement of Wnt/β-catenin signalling pathway in the regulation of cell migration and invasion is well known, we evaluated GSC invasive ability after treatment with 2 mM VPA for 96 h by Boyden chamber assay, performed on four cell lines. After treatment, GBM2 and G144 cell lines showed dramatic reductions of migrated cells (50% and 65%, respectively), while GBM04 and GBM7 cell lines displayed slightly increased values (Figure 4A).

We also investigated by Western Blot the VPA effect on Snail1 and Twist1 levels, two EMT activators, involved in the regulation of cell invasion.

Snail1 was significantly downregulated in GBM2 and G144 cell line (Figure 4B), while Twist1 was dowregulated only in GBM2. The other cell lines showed no variation or even an upregulation of these proteins (Figure 4B). Interestingly, the downregulation of Snail1 observed in GBM2 and G144 was correlated with the decrease of their invasive behaviour; conversely, the increase of protein levels seemed to match with the increase of the invasive ability observed in GBM7.

Finally, the evaluation of E-Cadherin and N-Cadherin expression after VPA treatment, by immunofluorescence, highlighted a relocalisation, especially of E-Cadherin, in the cell lines which showed an inhibition of their invasive behaviour (Figure 4C). N-Cadherin did not show any significant alterations. Interestingly, images pointed out also that all the GSCs underwent great morphological changes, confirming, once again, the effect of VPA on GSCs shape that we had previously detected [39].

## 4. Discussion

Epigenetic drugs such as HDACi are emerging as potential future anti-tumour therapies [6,7]. In particular, VPA appears to be one of the most promising HDACi, showing great anti-tumour effects in a wide range of cancers, including GBM [7,12,13], even if its exact mechanism of action is still not clear, due to the involvement of several biological processes and cell-signalling pathways. However, retrospective clinical studies reporting VPA’s capability of prolonging overall survival in GBM patients encouraged the study of this drug in the context of a future therapy for GBM treatment [9], the most common and aggressive primary brain tumour [1].

Starting from our previously-published results showing that VPA was able to induce a methylation change in multiple genes [7], we newly analysed those data by IPA software, to highlight any statistically-significant enrichment of genes involved in relevant molecular pathways. This analysis pointed out that multiple signalling pathways were affected by a VPA-induced methylation shift. We focused our attention on Wnt/β-catenin signalling, whose methylation status was found to have been altered by VPA in both the cell lines analysed, and whose role in GBM development and in GSCs regulation has already been demonstrated [23,24,25]. Intriguingly, the software outputs indicated that the Wnt/β-catenin pathway was potentially inhibited in GBM2, while it was potentially activated in G144 after treatment. However, these findings were only software previsions and needed further investigation.

Firstly, we evaluated the basal expression levels of seven Wnt/β-catenin pathway related genes (*WNT1*, *FZD4*, *CTNNB1*, *EP300*, *CREBBP*, *TCF7*, and *MYC*) and three Wnt specific target genes (*AXIN2*, *CD44*, *DKK1*) in seven GSC lines, showing that Wnt/β-catenin signalling pathway was not upregulated in GSCs compared to foetal neural stem cells.

Afterwards, we investigated the expression of the same genes after VPA treatment. Even if we observed a general downregulation of the transcriptional machinery of the pathway, represented by *CTNNB1, EP300*, and *CREBBP*, which can be due to the activation of a negative loop [40], target genes (except for *CD44* in GBM04) showed a strong upregulation after VPA exposure. This capability of VPA to induce an activation of the Wnt signalling pathway was confirmed by the increased transcriptional activity of the β-catenin/TCF complex. These results are in contrast to the data obtained by IPA analysis, which predicted an inactivation of the Wnt pathway in GBM2 after VPA treatment, strongly highlighting that predictive data always need experimental confirmation.

Intriguingly, the activation of Wnt/β-catenin was accompanied by a cell proliferation reduction in all the GSCs. This effect reminds us of our previously published data [39], and could be due, as already demonstrated, to VPA’s ability to modify the expression of genes involved in the cell cycle, stimulating p21 expression and G1/S block [41].

Since few studies have also elucidated that the Wnt/β-catenin pathway activation is able to enhance in vitro motility of GBM cells by activation of EMT [32], we next investigated whether the activation of the pathway induced by VPA could correlate with an effect on cell motility [24,42]. Surprisingly, the Boyden chamber assay revealed that VPA was able to induce a dramatic reduction of migrated cell number in two out of four cell lines (GBM2 and G144), whereas the other cell lines (GBM04 and GBM7) showed slightly-increased values in response to VPA exposure.

Hence, we also analysed the VPA effect on nuclear protein levels of Twist1 and Snail1, two transcription factors crucial for mesodermal differentiation in embryos and for the induction of EMT in cancers [43]. In fact, several studies highlighted that the same molecular players that play a key role in EMT of epithelial tumours are also involved in the regulation of motility of non-epithelial tumours, such as GBM [42]. Our results showed a strong decrease of the nuclear levels of Snail1 and Twist1 in GBM2, while in GBM7, both protein levels increased. Therefore, the opposite behaviour in migration ability seen in GBM7 and GBM2 was correlated with the respective increase or downregulation of the two EMT activators, while the decrease of the invasive behaviour observed in G144 cell line was correlated only with Snail1 downregulation. Hence, we might speculate that Snail1 is one of the most important molecular components in the regulation of migration behaviour in GSCs, whereas Twist1 might be a secondary player in this process.

In the GBM04 cell line, although it was not highlighted by any relevant variations in Twist1 and Snail1 protein levels, we observed an augmentation of the invasive behaviour that must be due to other mechanisms involved in invasion regulation, such as the involvement of the MMP-RECK, ERK, and JNK pathways [13].

Overall, these data suggested that VPA was selectively able to impair the activation of the EMT programme, through the downregulation of Twist1 and Snail1, but probably β-catenin was not the main mediator. In fact, even if the Wnt/β-catenin signalling pathway was activated in all the GSC lines, the latter showed a different response in terms of migration, suggesting the predominant involvement of other mechanisms in the regulation of this process. Moreover, in contrast with previous works, in our case, the activation of Wnt/β-catenin signalling pathway is not closely correlated with an upregulation of EMT factors or an enhanced GSC invasive behaviour [24], but, contrariwise, in some cell lines it was even accompanied by a downregulation of EMT factors and an inhibition of GSC migration. However, this VPA ambivalent action in modulating GSCs motility suggests a cell line-specific effect, which is likely deeply influenced by the molecular features of each cell line.

All these findings, linking the Wnt/β-catenin signalling pathway activation to an inhibitory effects of GSCs proliferation and migration, support previously published data, which highlighted the bivalent role of Wnt/β-catenin pathway, with its hyper-activation or repression both associated to pro- and anti-tumour effects, even in the same cancer type [30]. The role of Wnt/β-catenin activation has been demonstrated to be controversial also in brain tumours, with some studies showing that an increase in β-catenin levels can reduce glioma cell migration [44] and proliferation [45] and decrease stem cell markers expression [46], while others report that the overexpression of Wnt in glioma can promote GSC self-renewal and proliferation. Interestingly, Rampazzo and colleagues have also pointed out a Wnt/β-catenin-mediated reprogramming of GBM cells to a neuronal-like fate [47], suggesting that the activation of this signalling pathway is strongly involved in determining the pro-differentiative effect of VPA that we have previously reported [7]. The bi-valent role of the Wnt/β-catenin pathway can be a consequence of the heterogeneous genomic landscape of our GSC lines, as well as of the fact that many molecular pathways interact in order to modulate important cellular processes such as proliferation, migration, and invasion. Hence, our data feed the open question as to whether Wnt signalling needs to be therapeutically increased or decreased to achieve beneficial outcomes, pointing out that its activation could bring beneficial effects in the treatment of GBM.

In conclusion, all this evidence encourages the use of epigenetic drugs, such as VPA, in the context of a future therapy for the treatment of GBM, thanks to its pro-differentiative, anti-proliferative, and anti-migratory effects. However, investigations are certainly needed in order to deeply understand the GSC heterogeneous response, which leaves some open interrogatives, and animal models should be used to evaluate if the VPA action is effective within a tumour environment closer to a physiological one.

## Figures and Tables

**Table 1:** Fold regulation of the expression variation of 84 Wnt signalling pathway-related genes. The Real-Time PCR was performed on GBM2 and G144 cell lines after 96 h of exposure to 2 mM VPA. Downward arrows indicate gene downregulation (values < −1.5), upward arrows indicate gene upregulation (values > 1.5), while the equal sign means that no transcriptional changes were detected. The genes that showed the same alteration or no alterations in both the cell lines after VPA exposure are written in bold.

Genes	GBM2	G144	Genes	GBM2	G144	Genes	GBM2	G144
*AES*	↓	↓	*FRAT1*	=	=	*SFRP1*	↑	↓
*APC*	=	=	*FRZB*	=	↑	*SFRP4*	↑	↑
*AXIN1*	↓	↓	*FSHB*	↑	↑	*FBXW4*	↓	↓
*BCL9*	=	=	*FZD1*	↑	↓	*SLC9A3R1*	↓	↓
*BTRC*	=	↓	*FZD2*	=	↓	*SOX17*	↑	↑
*CCND1*	↑	↓	*FZD3*	=	↓	*T*	↑	↑
*CCND2*	↑	↓	*FZD4*	↑	↑	*TCF7*	↑	↑
*CCND3*	↑	↓	*FZD5*	=	=	*TCF7L1*	=	↑
*CSNK1A1*	=	↓	*FZD6*	=	↓	*TLE1*	↑	↑
*CSNK1D*	=	↓	*FZD7*	=	↓	*TLE2*	=	↓
*CSNK1G1*	=	↑	*FZD8*	↑	↓	*WIF1*	↑	↑
*CSNK2A1*	=	↓	*GSK3A*	↓	↓	*WISP1*	=	↑
*CTBP1*	=	↓	*GSK3B*	↑	=	*WNT1*	↑	↑
*CTBP2*	=	=	*JUN*	=	↓	*WNT10A*	↑	↑
*CTNNB1*	=	=	*KREMEN1*	=	↓	*WNT11*	↓	↑
*CTNNBIP1*	=	↑	*LEF1*	=	↓	*WNT16*	↓	↑
*CXXC4*	↑	↑	*LRP5*	↓	↓	*WNT2*	↑	↑
*DAAM1*	↑	=	*LRP6*	=	↓	*WNT2B*	=	=
*DIXDC1*	↑	↑	*MYC*	↑	↓	*WNT3*	↓	↓
*DKK1*	↑	↑	*NKD1*	↓	↑	*WNT3A*	↑	↑
*DVL1*	↑	=	*NLK*	↑	↓	*WNT4*	↑	=
*DVL2*	=	↑	*PITX2*	=	↓	*WNT5A*	↑	↓
*EP300*	=	=	*PORCN*	=	↓	*WNT5B*	↑	=
*FBXW11*	=	=	*PPP2CA*	↑	↓	*WNT6*	↑	↑
*FBXW2*	↑	↑	*PPP2R1A*	=	↓	*WNT7A*	↑	↑
*FGF4*	↑	↑	*PYGO1*	=	↑	*WNT7B*	↑	↓
*FOSL1*	↑	=	*RHOU*	↑	=	*WNT8A*	↑	↑
*FOXN1*	=	*↑*	*SENP2*	=	*↓*	*WNT9A*	*↓*	*↑*

## Supplementary Materials

The following are available online at http://www.mdpi.com/2073-4425/9/11/522/s1. Figure S1: IPA analysis of GBM2 genome-wide DNA methylation status data. Several pathways have emerged from the IPA analysis as affected by changes in methylation status after 96 h exposure to VPA 2 mM. Pathways are reported from the most to the less statistically significant. Black arrow indicates Wnt signalling pathway. Figure S2: IPA analysis of G144 genome-wide DNA methylation status data. (A) Several pathways have emerged from the IPA analysis as affected by changes in methylation status after 96 h exposure to VPA 2 mM. Pathways are reported from the most to the less statistically significant. Black arrow indicates Glioblastoma signalling pathway, which includes Wnt signalling pathway. (B) Representative image of Glioblastoma multiforme signalling pathway in 96 h treated G144 cells obtained from IPA software confirmed the inclusion of the Wnt signalling pathway in the most general category of Glioblastoma signalling pathway. Green = gene potentially expressed (unmethylated status after treatment); Red = gene potentially unexpressed (methylated status after treatment). Table S1: Primers used for Real Time PCR. Table S2: Copy number alterations of selected Wnt signalling pathway-related genes detected in six GSC lines. Numbers in table represent copy number alterations (CNAs), which were calculated using the following formula: CN =$\;2\cdot 2^{\log_{2}ratio}$. CN = 2 (−) indicates no copy number alterations compared to reference control, CN > 2 indicates copy number gain, CN < 2 indicates copy number loss. Table S3: Statistical analysis (*p*-values, *t*-test) of the effects of VPA on cell metabolic activity. *p*-values are referred to the specific treatment compared to the respective untreated cells. ns = not statistically significant.
