# Supplementary material for: Valproic Acid Inhibits Proliferation and Reduces Invasiveness in Glioma Stem Cells Through Wnt/β Catenin Signalling Activation
Source: Genes (Basel). 2018 Oct 26;9(11):522. doi: 10.3390/genes9110522 (PMC6267016; doi:10.3390/genes9110522)
Supplement: Supplementary file 1 [file genes-09-00522-s001.zip › genes-367416_supplementary1/Table S1.docx]

**Table S1. Primers used for Real Time PCR**

| **Gene** | **Forward 5’->3’** | **Reverse 5’->3’** |
| --- | --- | --- |
| *WNT1* | CTGGCTGGGTTTCTGCTAC | GAGGAGGCTACGTTCACAATAC |
| *FZD4* | TACCTCACAAAACCCCCATCC | GGCTGTATAAGCCAGCATCAT |
| *CTNNB1* | CATCCTAGCTCGGGATGTTCAC | TCCTTGTCCTGAGCAAGTTCAC |
| *EP300* | TCCGAGACATCTTGAGACGACAG | GGGTTGCTGGAACTGGTTATGG |
| *CREBBP* | TGGAAGACCGAGTGA ACAA | GTGGGTGGCAATGGAAGA |
| *TCF7* | GAGTCACTGTCCATGTCTTCT | TATGGGCTGTCATCTGTCTTC |
| *MYC* | CCTGGTGCTCCATGAGGAGA | CAGTGGGCTGTGAGGAGGTTT |
| *AXIN2* | AGCCAAAGCGATCTACAAAAGG | GGTAGGCATTTTCCTCCATCAC |
| *CD44* | GTGATGGCACCCGCTATG | ACTGTCTTCGTCTGGGATGG |
| *DKK1* | CCTTGAACTCGGTTCTCAATTCC | CAATGGTCTGGTACTTATTCCCG |
