# Supplementary material for: Valproic Acid Inhibits Proliferation and Reduces Invasiveness in Glioma Stem Cells Through Wnt/β Catenin Signalling Activation
Source: Genes (Basel). 2018 Oct 26;9(11):522. doi: 10.3390/genes9110522 (PMC6267016; doi:10.3390/genes9110522)
Supplement: Supplementary file 1 [file genes-09-00522-s001.zip › genes-367416_supplementary1/Table S2.docx]

**Table S2. Copy number alterations of selected Wnt signaling pathway-related genes detected in six GSC lines.** Numbers in table represent copy number alterations (CNAs), which were calculated using the following formula: CN =${2\cdot2}^{\log_{2} ratio}$. CN=2 (-) indicates no copy number alterations compared to reference control, CN>2 indicates copy number gain, CN<2 indicates copy number loss.

| **Genes** | **Cytoband** | **GBM2** | **GBM7** | **G144** | **G166** | **G179** | **GliNS2** |
| --- | --- | --- | --- | --- | --- | --- | --- |
| APC | 5q22.2 | - | - | - | - | 1.40 | - |
| AXIN1 | 16p13.3 | - | 2.97 | - | - | 2.50 | - |
| AXIN2 | 17q24.1 | - | - | - | - | - | - |
| CCND1 | 11q13.3 | 1.41 | 1.26 | - | - | - | - |
| CMYC | 8q24.21 | - | - | - | - | - | - |
| CREBBP | 16p13.3 | - | 2.66 | - | - | 2.50 | - |
| CSNK1A1 | 5q32 | - | - | - | - | 1.30 | - |
| CSNK2A1 | 20p13 | - | - | - | - | - | - |
| CTNNB1 | 3p22.1 | - | - | - | - | - | - |
| CTNNBIP1 | 1p36.2 | - | - | - | 2.60 | 1.44 | 1.01 |
| DKK1 | 10q21.1 | - | - | - | - | - | - |
| EP300 | 22q13.2 | 2.91 | 3.21 | - | - | - | - |
| FAT1 | 4q35.2 | - | - | - | - | - | - |
| FOXM1 | 12p13.33 | - | - | - | - | - | - |
| FZD2 | 17q21.31 | - | - | - | - | - | - |
| FZD4 | 11q14.2 | - | - | - | - | - | - |
| FZD6 | 8q22.3 | - | - | - | - | - | - |
| FZD7 | 2q33.1 | - | - | - | - | - | - |
| GSK3β | 3q13.33 | - | - | - | 3.12 | - | - |
| LEF1 | 4q25 | 1.33 | - | - | - | 1.57 | - |
| SFRP1 | 8p11.21 | - | - | - | - | - | - |
|  |  |  |  |  |  |  |  |
| SFRP2 | 4q31.3 | - | - | - | - | - | - |
| SFRP4 | 7p14.1 | - | - | - | - | - | - |
| SFRP5 | 10q24.2 | - | - | - | - | - | - |
| TCF4 | 18q21.2 | 1.42 | - | - | - | - | - |
| TCF7 | 5q31.1 | - | - | - | 3.12 | 1.33 | - |
| WIF1 | 12q14.3 | - | - | 1.07 | 2.39 | - | - |
| WNT1 | 12q13.12 | - | - | - | - | - | - |
| WNT2 | 7q31.2 | - | - | - | - | 2.93 | 3.16 |
| WNT5A | 3p14.3 | 1.41 | - | - | 2.91 | - | - |
