# Supplementary material for: Valproic Acid Inhibits Proliferation and Reduces Invasiveness in Glioma Stem Cells Through Wnt/β Catenin Signalling Activation
Source: Genes (Basel). 2018 Oct 26;9(11):522. doi: 10.3390/genes9110522 (PMC6267016; doi:10.3390/genes9110522)
Supplement: Supplementary file 1 [file genes-09-00522-s001.zip › genes-367416_supplementary1/Table S3.docx]

**Table S3. Statistical analysis (*p*-values, t-test) of the effects of VPA on cell metabolic activity**. *p*-values are referred to the specific treatment compared to the respective untreated cells. ns = not statistically significant

|  | VPA 96hs | | |
| --- | --- | --- | --- |
|  | **0.5 mM** | **1 mM** | **3 mM** |
| GBM04 | 0.0001 | 0.0001 | 0.0001 |
| GBM2 | 0.0001 | 0.0001 | 0.0001 |
| GBM7 | ns | ns | 0.01 |
| G144 | ns | ns | ns |
| G179 | 0.0001 | 0.0001 | 0.0001 |
| GLINS2 | 0.001 | 0.001 | 0.0001 |
| G166 | ns | 0.01 | 0.05 |
