# Supplementary figures and images for: Valproic Acid Inhibits Proliferation and Reduces Invasiveness in Glioma Stem Cells Through Wnt/β Catenin Signalling Activation
Source: Genes (Basel). 2018 Oct 26;9(11):522. doi: 10.3390/genes9110522 (PMC6267016; doi:10.3390/genes9110522)

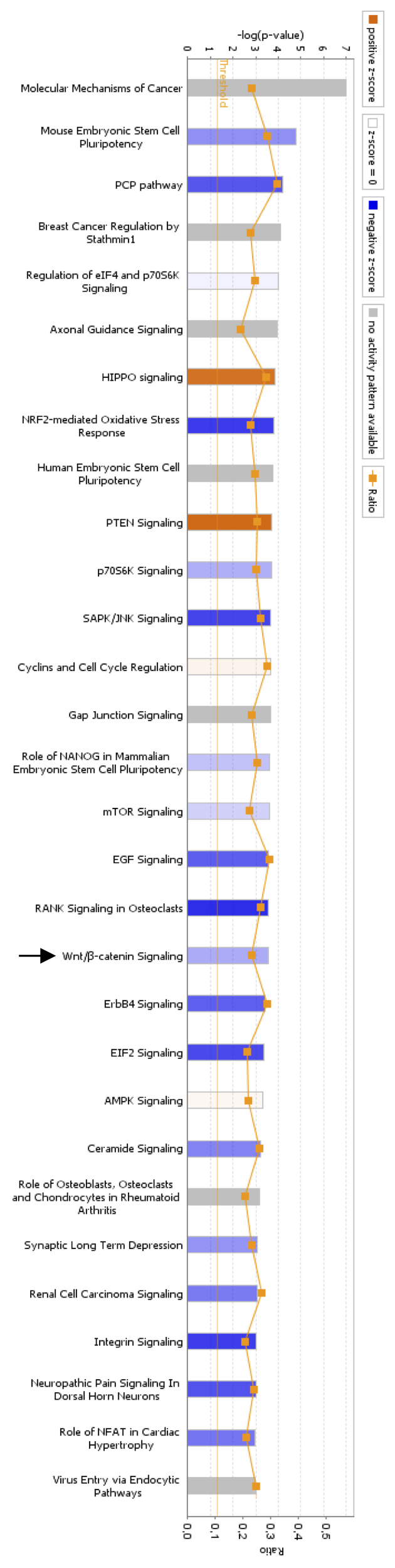

Supplement: Supplementary file 1 [file genes-09-00522-s001.zip › genes-367416_supplementary1/Figure S1.jpg]

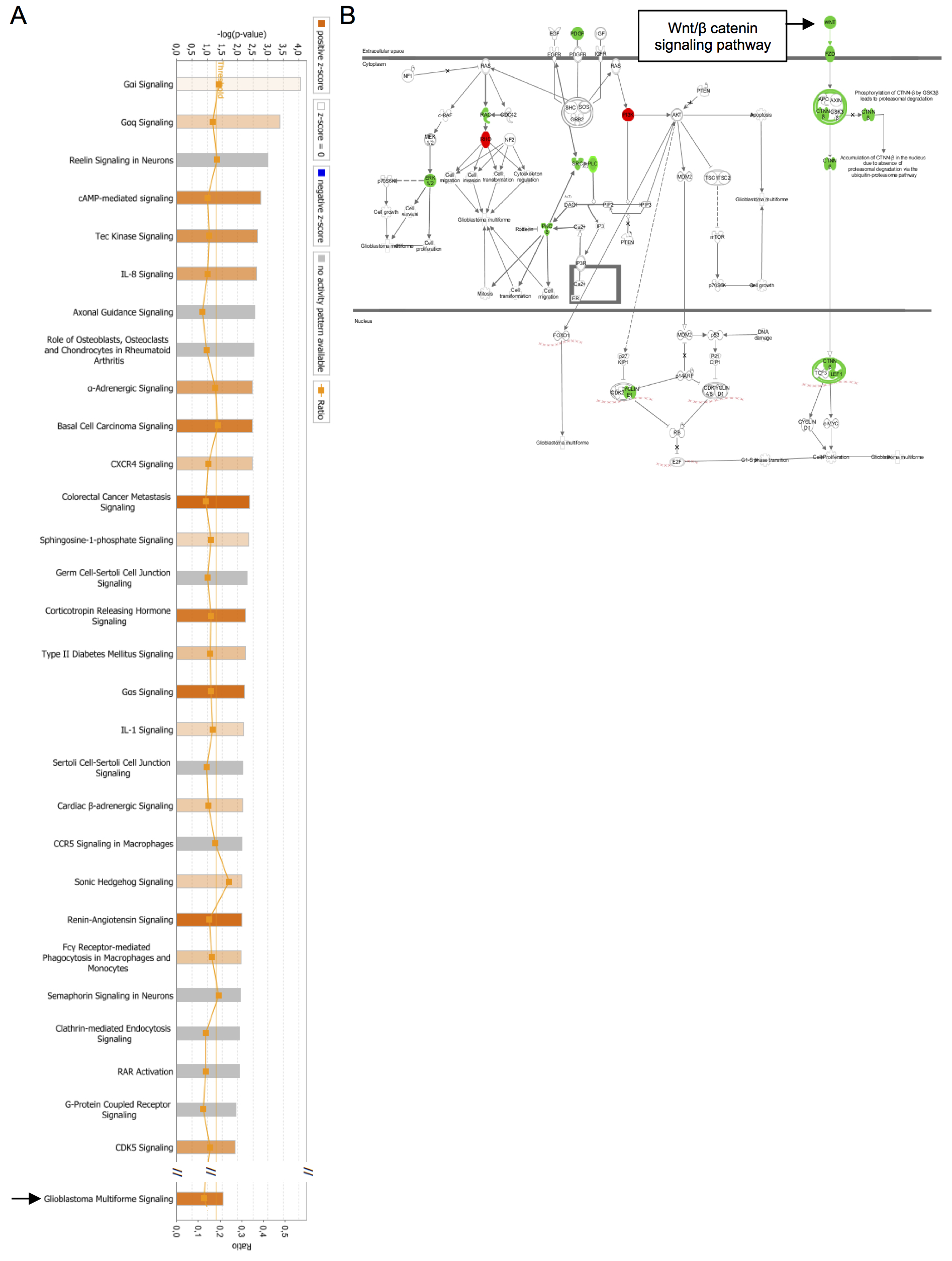

Supplement: Supplementary file 1 [file genes-09-00522-s001.zip › genes-367416_supplementary1/Figure S2.jpg]
